# Correction: KEAP1 loss modulates sensitivity to kinase targeted therapy in lung cancer

**DOI:** 10.7554/eLife.33173

**Published:** 2017-10-31

**Authors:** Elsa Beyer Krall, Belinda Wang, Diana M Munoz, Nina Ilic, Srivatsan Raghavan, Matthew J Niederst, Kristine Yu, David A Ruddy, Andrew J Aguirre, Jong Wook Kim, Amanda J Redig, Justin F Gainor, Juliet A Williams, John M Asara, John G Doench, Pasi A Janne, Alice T Shaw, Robert E McDonald III, Jeffrey A Engelman, Frank Stegmeier, Michael R Schlabach, William C Hahn

Krall BE, Wang B, Munoz DM, Ilic N, Raghavan S, Niederst MJ, Yu K, Ruddy DA, Aguirre AJ, Kim JW, Redig AJ, Gainor JF, Williams JA, Asara JM, Doench JG, Janne PA, Shaw AT, McDonald III RE, Engelman JA, Stegmeier F, Schlabach MR, Hahn WC. 2017. KEAP1 loss modulates sensitivity to kinase targeted therapy in lung cancer. *eLife* **6**:e18970. doi: 10.7554/eLife.18970.

Published 1, February 2017

In the published article, there is an error in the inline table for sgRNA sequences—the “Target sequence” for sgKEAP1-4is shown as “GAGGACACACTTCTCGCCCA”, which is a duplicate of the target sequence for sgKEAP1-3. The correct “Target sequence” for sgKEAP1-4is ACTGGGCGGCCGGTGCATCC.

The Corrected Table is shown here:

sgRNA Sequences

**Table:** 

Name	Target Sequence
sgGFP	GGCGAGGGCGATGCCACCTA
sgKEAP1-1	CTTGTGGGCCATGAACTGGG
sgKEAP1-2	TGTGTCCTCCACGTCATGAA
sgKEAP1-3	GAGGACACACTTCTCGCCCA
sgKEAP1-4	ACTGGGCGGCCGGTGCATCC
sgLACZ-1	AACGGCGGATTGACCGTAAT
sgLACZ-2	CTAACGCCTGGGTCGAACGC

The originally published Table is also shown for reference:

sgRNA Sequences

**Table:** 

Name	Target Sequence
sgGFP	GGCGAGGGCGATGCCACCTA
sgKEAP1-1	CTTGTGGGCCATGAACTGGG
sgKEAP1-2	GAGGACACACTTCTCGCCCA
sgKEAP1-3	GAGGACACACTTCTCGCCCA
sgKEAP1-4	GAGGACACACTTCTCGCCCA
sgLACZ-1	AACGGCGGATTGACCGTAAT
sgLACZ-2	CTAACGCCTGGGTCGAACGC

The article has been corrected accordingly.

